# Special Issue “GPCR: Roles in Cell Development and Disease”

**DOI:** 10.3390/ijms24097943

**Published:** 2023-04-27

**Authors:** Roberta Lattanzi, Rossella Miele

**Affiliations:** 1 Department of Physiology and Pharmacology “Vittorio Erspamer”, Sapienza University of Rome, Piazzale Aldo Moro 5, I-00185 Rome, Italy; 2 Department of Biochemical Sciences “A. Rossi Fanelli”, Sapienza University of Rome, Piazzale Aldo Moro 5, I-00185 Rome, Italy

We are pleased to present the following Special Issue of the *International Journal of Molecular Sciences (IJMS)*, entitled “GPCR: Roles in Cell Development and Disease”.

G-protein-coupled receptors (GPCRs), the most abundant group of human cell surface receptors, are physiologically important for the maintenance of homeostasis, particularly through their ability to mediate responses to circulating hormones and neurotransmitter inputs from the central nervous system to peripheral organs.

The goal of this Special Issue is to review new aspects of GPCR activation and signaling during cell development, including the morphogenetic processes that drive the formation of highly specialized organs. Once development is complete, some signaling pathways are maintained to ensure adult tissue homeostasis. In addition, morphogenetic pathways that are important for development are altered by invasive cells in tumor metastases and a variety of other diseases of genetic origin. Further insights into the biochemical, pharmacological, and structural evidence for the molecular mechanisms of GPCR activation, which play key roles in various physiological processes in every major organ system, including the CNS and cardiovascular, respiratory, metabolic, and urogenital systems, may provide new molecular targets for the development of new, highly specific drugs with fewer side effects in clinical medicine [1].

Prokineticins are a new class of chemokine-like peptides that bind to their GPCRs, PKR1 and PKR2, and play functional roles in numerous physiological processes, including intestinal contraction, circadian rhythms, vascular and reproductive functions, and pathological conditions such as inflammatory responses and cancer [2,3,4,5].

Prokienticin receptor 2 (PKR2) plays a critical role in olfactory bulb development (OB). Both PKR2 and its endogenous ligand prokineticin 2 (PK2) are secreted in OB and act as chemoattractants for neuronal precursors from the subventricular zone (SVZ).

In mice lacking the Pkr2 and Pk2 genes, OB is smaller and exhibits an altered architecture. There is also an accumulation of neuronal progenitor cells, resulting in a smaller number of GnRH neurons in the hypothalamus. The failure of GnRH secretion is associated with low plasma levels of testosterone and FSH and impaired sexual development. Similarly, female PKR2 and PK2 knockout mice show impaired estrous cycles. Human mutations of PKR2 and PK2 are associated with idiopathic hypogonadotropic hypogonadism (IHH) and Kallmann syndrome (KS) [3].

The accessory melanocortin receptor protein 2 (MRAP2) is a small transmembrane protein arranged in a double antiparallel topology on the plasma membrane. It is expressed almost exclusively in the paraventricular nucleus of the hypothalamus, where it interacts with several GPCRs and regulates energy expenditure and appetite. In particular, it increases food intake after interaction with, and inhibition of, PKR2 [6,7].

In the first paper included in this Special Issue, the authors analyzed the functional role of the specific arginine residue at position 125 of MRAP2. For this purpose, the authors prepared two MRAP2 mutants in which the Arg125 residue was replaced with histidine (R125H) or cysteine (R125C), which are present in human patients with extreme obesity. They analyzed their interactions with PKR2 and defined the minimal C-terminal domain region of MRAP2 as sufficient to ensure dimer formation. They also analyzed PKR2 activation in the presence of the MRAP2 mutants and assessed the ERK pathway’s activation induced by PK2. The study showed that R125H reduced PK2 activation more effectively than R125C and the MRAP2 wild type. These results are particularly interesting because they show that the Arg125 mutation affects MRAP2 conformation, dimer formation propensity, and PKR2 binding capacity. The study of the structure–function relationship aids in the development of new drugs that promote the dissociation of the MRAP2-PKR2 complex and specifically block the given signaling pathway [8].

In the other studies: the authors used an advanced form of fluorescence spectroscopy, Linescan Fluorescence Correlation Spectroscopy (FCS), to observe how the levels of GPCRs and β1- and β2-adrenergic receptors (β1/2-ARs), labeled with a bright and photostable fluorescent antagonist, changed during the long-term monolayer culture of cardiomyocytes (CMs) derived from human induced pluripotent stem cells (hiPSCs). They compared the kinetics of β1/2-ARs mRNA levels observed in CMs with β1/2-ARs mRNA levels observed in wild-type (WT) hiPSCs and in two CRISPR/Cas9 knock-in clones. This is an important study that deepens our understanding of how the membrane localization of CM-specific GPCRs evolves during differentiation [9].
